# A placebo-controlled study of the doses and efficacy of *Lentinula edodes* mycelia for oxaliplatin-induced peripheral neuropathy in colorectal cancer

**DOI:** 10.3389/fonc.2025.1577848

**Published:** 2025-08-11

**Authors:** Shogen Boku, Hironaga Satake, Seiichiro Mitani, Kiyoshi Maeda, Toshihiro Kudo, Toshifumi Yamaguchi, Tatsuya Takagi, Hisato Kawakami

**Affiliations:** ^1^ Department of Clinical Oncology, Kansai Medical University Hospital, Osaka, Japan; ^2^ Department of Medical Oncology, Kochi Medical School, Kochi, Japan; ^3^ Department of Medical Oncology, Kindai University Faculty of Medicine, Osaka, Japan; ^4^ Department of Gastroenterological Surgery, Osaka Metropolitan University Graduate School of Medicine, Osaka, Japan; ^5^ Department of Medical Oncology, Osaka International Cancer Institute, Osaka, Japan; ^6^ Cancer Chemotherapy Center, Osaka Medical and Pharmaceutical University, Osaka, Japan; ^7^ Graduate School of Pharmaceutical Sciences, Osaka University, Osaka, Japan; ^8^ Department of Clinical Oncology, Tohoku University Graduate School of Medicine, Miyagi, Japan

**Keywords:** chemotherapy-induced peripheral neuropathy, colorectal cancer, *Lentinula edodes* mycelia, L.E.M., oxaliplatin

## Abstract

**Introduction:**

Preclinical studies have demonstrated the potential of *Lentinula edodes* mycelium (L.E.M.) extract for managing oxaliplatin-induced peripheral neuropathy (OIPN). The efficacy and optimal dosage of L.E.M. for OIPN remain uncertain. We evaluated the efficacy and safety as well as the optimal dosage of L.E.M. extract for OIPN in patients with colorectal cancer (CRC).

**Methods:**

After curative resection, we used a 1:1:1 ratio to randomly assign patients with CRC with persistent OIPN (defined by a visual analogue scale [VAS] numbness score ≥40 mm) to the low-dose (L.E.M. 300 mg twice daily [bid]), high-dose (L.E.M. 900 mg bid), and placebo groups. The primary endpoint of this double-blind, placebo-controlled phase 2 trial was the reduction in the VAS numbness score in the low-dose group compared with that in the placebo group at 12 weeks. Adverse events (AEs) and quality of life were evaluated.

**Results:**

Forty-five patients were randomly assigned to the placebo (n = 15), low-dose (n = 15), and high-dose (n = 15) groups. At 12 weeks, no significant difference in the reduction of the VAS numbness score was observed when the low-dose and placebo groups were compared (−12.20 [95% confidence interval {CI}; −34.54 to 10.14] *vs*. −10.69 [95% CI; −27.07 to 5.69]; p = 0.83); however, the high-dose group showed a favorable trend compared with the placebo group (−12.20 *vs*. −29.32 [95% CI; −53.4 to −5.2]; p = 0.06). Grade 3 or higher AEs related to the intervention were not observed.

**Discussion:**

High-dose L.E.M. extract resulted in a clinically meaningful improvement in VAS numbness scores without severe toxicity.

**Clinical Trial Registration:**

https://jrct.mhlw.go.jp/en-latest-detail/jRCTs051210085, identifier jRCTs051210085.

## 1 Introduction

Chemotherapy-induced peripheral neuropathy (CIPN) is a common and cumulative side effect of anticancer drugs such as oxaliplatin, paclitaxel, vincristine, and bortezomib (1). Symptoms include extremity numbness, sensory ataxia, decreased deep tendon reflexes, and muscle weakness (2). These symptoms can severely impact patients’ quality of life (QoL) by causing gait disturbances and hindering daily activities as well as limiting the continuation of chemotherapy. Currently, the combination of 5-fluorouracil (5-FU) and oxaliplatin is the standard adjuvant chemotherapy for locally advanced colorectal cancer (high-risk stage II or stage III), as supported by multiple phase III trials (3–5). Although this regimen improves survival, it is associated with cumulative peripheral neuropathy, which is a well-known adverse event associated with oxaliplatin. Prolonged oxaliplatin use progressively worsens neuropathy. Studies have reported that persistent oxaliplatin-induced peripheral neuropathy (OIPN) was observed in up to 15% of patients at 4 years post-treatment (6). Because no effective agents other than duloxetine for the treatment of oxaliplatin-induced numbness have been identified (7, 8), reducing the chemotherapy duration from 6 months to 3 months is a viable option for patients with high-risk stage II or low-risk stage III disease (9). However, for patients with high-risk disease, 6 months of therapy remains the standard; therefore, OIPN is an ongoing clinical challenge.

*Lentinula edodes* mycelium (L.E.M.) extract is produced by inoculating shiitake mushroom fungi into a solid medium primarily composed of bagasse and rice bran, cultivating the fungi for several months until just before mushroom development, and subsequently extracting the fungi with boiling water (10). Previous studies suggested that L.E.M. extract may help maintain patients’ QoL, primarily by preventing chemotherapy-related taste disorders. Thus, L.E.M. extract has shown potential benefits for patients with cancer (11, 12). However, although L.E.M. may improve the QoL of patients with cancer and is safe for long-term use at high doses, its efficacy and optimal dosage for OIPN remained uncertain (10, 11, 13). To address this uncertainty, we conducted a double-blind, placebo-controlled study to evaluate the efficacy and safety of L.E.M. extract for OIPN in patients undergoing postoperative chemotherapy for colorectal cancer (CRC) (10).

## 2 Materials and methods

### 2.1 Study enrolment

Participants were recruited from six institutes in Japan. Eligible individuals were 20 years of age or older, were diagnosed with grade 1 or higher peripheral sensory neuropathy (according to the Common Terminology Criteria for Adverse Events [CTCAE] version 4.0) and had a visual analogue scale (VAS) numbness score of 40/100 mm or higher at least 3 months after completing oxaliplatin treatment. Participants were required to have pathologically confirmed CRC following curative resection, an Eastern Cooperative Oncology Group performance status of 0 to 2, adequate major organ function, and the ability to use oral medications. Additionally, participants were required to write with their dominant hand during the VAS assessment. Patients using gabapentin were excluded because L.E.M. extract may affect blood levels. Concomitant use of anti-CIPN medications other than gabapentin was allowed if the patient had been using stable doses for at least 2 weeks prior to randomization. Specifically, no new anti-CIPN agents could be initiated, existing anti-CIPN medications could not be discontinued, and the total dose of anti-CIPN medication could not increase or decrease by more than 10% during the 2 weeks prior to study enrolment.

This study was conducted in accordance with the principles of the Declaration of Helsinki and the Japanese Clinical Trials Act. Approval was granted by the Clinical Research Review Board of Nara Medical University (CRB5200002), and this study was registered in the Japanese Clinical Trials Registry (jRCTs051210085). All patients provided written informed consent before participation.

### 2.2 Intervention

Participants were randomized using a 1:1:1 ratio to the placebo, low-dose, and high-dose groups. Randomization was performed using dynamic allocation (minimization method). A list of study product numbers corresponding to the target sample size was generated using an Excel-based random number table. These numbers were randomly assigned to three groups. A study product manager who was not involved in the conduct of the study attached the study product numbers to each product package. The assignment table linking the study product numbers and groups was securely stored and accessed only at the time of unblinding. At the time of subject registration, investigators communicated the subject’s allocation factors (age, gender, and numbness score assessed by VAS) to the allocation center. Based on these factors and the already registered subjects’ allocations, the allocation center performed dynamic allocation. The center then reported the assigned study product numbers to the investigators, who administered the assigned treatment to the subjects. The low-dose group received 600 mg/day (300 mg twice daily) and the high-dose group received 1800 mg/day (900 mg twice daily) of L.E.M. extract-containing compounds for 12 weeks. The protocol treatment was discontinued if a causal relationship with L.E.M. could not be denied or if deemed necessary by the participant or investigator.

### 2.3 Measurements

The VAS score was rated using a 100-mm horizontal line. Participants were informed that the left end of the scale represented ‘no pain’ and that the right end represented ‘the most intense pain imaginable’. They were asked to mark the intensity of their current pain on the scale (14). Patient-reported OIPN-related QoL was assessed using the Functional Assessment of Cancer Therapy-Gynecologic Oncology Group-Neurotoxicity (FACT/GOG-Ntx) questionnaire, which was designed to assess CIPN severity, including sensory, motor, and functional impairment, and its impact on QoL (15, 16). One systematic review found evidence supporting the use of the FACT/GOG-Ntx to assess CIPN in the research setting (17). The FACT/GOG-Ntx comprises 11 items related to neurotoxicity, with each rated using a 5-point scale (range, 0–4). FACT/GOG-Ntx scores range from 0 to 44, with lower scores indicating lower neurotoxicity. Any adverse events, including sensory neuropathy, were evaluated based on the criteria of CTCAE version 4.0.

### 2.4 Study design and endpoints

This study was a three-arm, randomized, double-blind, placebo-controlled phase II trial that evaluated the efficacy of low-dose and high-dose L.E.M. extract to reduce OIPN severity in patients with CRC. The primary endpoint was the magnitude of change in the VAS numbness score in the low-dose group compared with that in the placebo group after 12 weeks of treatment. Secondary endpoints were the magnitude of change in VAS score for pain in the placebo group compared with that in the low-dose group at 12 weeks, that for numbness and pain in the placebo compared with that in the high-dose group at 12 weeks, and that for numbness among all groups at 4 weeks and 8 weeks; CTCAE grades of peripheral sensory neuropathy of all groups at 4 weeks; QoL scores of all groups according to the FACT/GOG-Ntx; and the incidence of adverse events of all groups.

### 2.5 Sample size

Previous clinical trial reports of the efficacy of duloxetine for Japanese patients with anticancer drug-induced peripheral neuropathy showed a mean VAS score change of -26.0 in the duloxetine group compared with -8.0 in the control group administered vitamin B12 after 4 weeks of treatment (18). Based on these results, the expected mean change in the VAS score for peripheral neuropathy after 12 weeks for the placebo group was set at -8.0, and the target mean change in the low-dose group was set at -36.0 with a standard deviation of 25.0. Using these parameters, we calculated that a sample size of 14 patients per group was necessary to detect a statistically significant difference in VAS score changes between the groups with a power of 80% and significance level of 5%. To account for potential dropouts and ineligible cases, we increased the target number of patients to 15 per group, which ensured that the study would remain adequately powered even if some participants did not complete the study or were excluded from the final analysis.

### 2.6 Statistical analysis

VAS scores and CTCAE grades in the low-dose, high-dose, and placebo groups were compared using two-tailed t-tests. Changes in FACT/GOG-Ntx QoL scores were evaluated using the Mann-Whitney-Wilcoxon test. The incidence of adverse events was compared using Fisher’s exact test. All statistical analyses were performed using SPSS (IBM version 26).

## 3 Results

### 3.1 Participant characteristics

**Figure 1:**
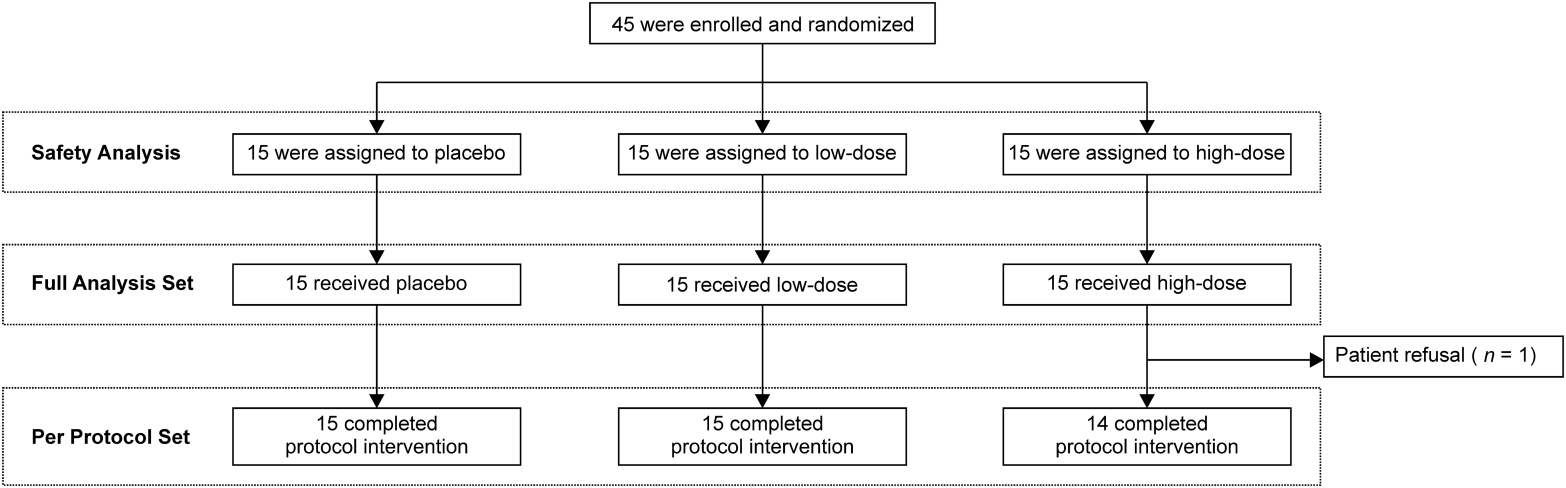
CONSORT diagram.

A total of 45 patients were enrolled between September 2021 and June 2023 (**Figure 1**). Patients were randomly assigned to the placebo (n = 15), low-dose (n = 15), and high-dose (n = 15) groups. One patient in the high-dose group discontinued treatment at week 3 because of personal reasons for refusal; therefore, that patient was excluded from the per-protocol analysis. The baseline characteristics of each group were broadly similar to those of the overall population (**Table 1**). The mean ages in the placebo, low-dose, and high-dose groups were 63.7 years, 65.5 years, and 67.9 years, respectively. The proportions of male patients in the placebo, low-dose, and high-dose groups were 66.7%, 66.7%, and 53.3%, respectively. The proportion of patients with an Eastern Cooperative Oncology Group performance status of 0 was 73.3%, 86.7%, and 66.7% in the placebo, low-dose, and high-dose groups, respectively. CTCAE grade of peripheral neuropathy 1 was observed in 40.0%, 26.7%, and 26.7% of patients in the placebo, low-dose, and high-dose groups, respectively. The median times from diagnosis to enrolment in the placebo, low-dose, and high-dose groups were 460 days (range, 134–1067), 335 days (range, 126–1921), and 470 days (range, 128–3366).

**Table 1:** Patient characteristics.

Characteristics	Placebo group (*n*=15)	Low-dose group (*n*=15)	High-dose group (*n*=15)
Age, mean (median, range), years	63.7 (59, 54–85)	65.5 (68, 49–80)	67.9 (72, 46–78)
Sex (%)			
Male	10 (66.7)	10 (66.7)	8 (53.3)
Female	5 (33.3)	5 (33.3)	7 (46.7)
ECOG PS (%)			
0	11 (73.3)	13 (86.7)	10 (66.7)
1	4 (26.7)	2 (13.3)	5 (33.3)
CTCAE grade of peripheral neuropathy (%)			
1	6 (40.0)	4 (26.7)	4 (26.7)
2	8 (53.3)	10 (66.7)	7 (46.7)
3	1 (6.7)	1 (6.7)	4 (26.7)
Time between diagnosis and enrollment, median (range), days	460 (134–1067)	335 (126–1921)	470 (128–3366)

CTCAE, Common Terminology Criteria for Adverse Events; ECOG PS, Eastern Cooperative Oncology Group performance status.

### 3.2 VAS-based outcome measures

**Figure 2:**
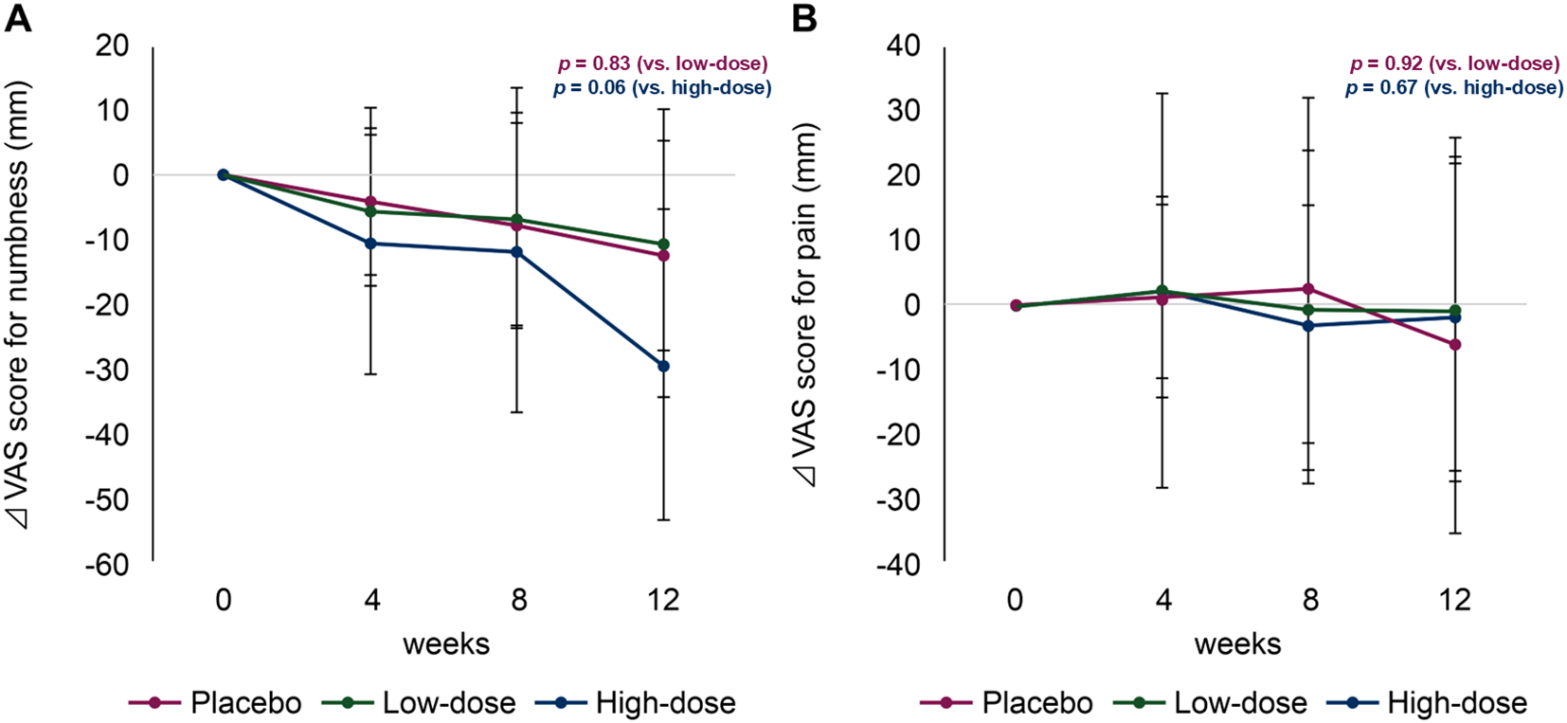
Changes in visual analogue scale (VAS) scores for numbness **(A)** and pain **(B)**. Magenta line, placebo group; green line, low-dose group; blue line, high-dose group.

A significant difference in the reduction of the VAS numbness scores in the low-dose and placebo groups at 12 weeks was not observed (−12.20 [95% confidence interval {CI}, −34.54 to 10.14] *vs*. −10.69 [95% CI, −27.07 to 5.69]; *p* = 0.83) (**Figure 2A**). Detailed changes in VAS scores are summarized in **Table 2**. However, the high-dose group showed a greater, albeit not statistically significant, improvement in the VAS numbness score than the placebo group (−12.20 *vs*. −29.32 [95% CI, −53.35 to −5.29]; *p* = 0.06) (**Figure 2B**). No significant differences were observed when the VAS pain scores in the low-dose and placebo groups were compared (−1.83 [95% CI, −25.56 to 21.90] *vs*. −0.90 [95% CI, −27.51 to 25.71]; *p* = 0.92), or when comparing the high-dose and placebo groups (−6.05 [95% CI, −23.06 to 10.96]; *p* = 0.67).

**Table 2:** Visual analog scale (VAS) scores for numbness and pain.

Outcome	Timepoint	Placebo (*n*=15)	Low-dose group (*n*=15)	High-dose group (*n*=14)	*P*-value (placebo *vs*. low-dose groups)	*P*-value (placebo *vs*. high-dose groups)
Numbness, mean ± SD, mm	Baseline	60.81 ± 16.77	65.74 ± 16.73	67.01 ± 13.78		
	4 weeks	57.96 ± 15.93	60.63 ± 20.58	56.03 ± 18.06		
	8 weeks	53.22 ± 18.12	58.85 ± 24.32	55.31 ± 23.10		
	12 weeks	48.61 ± 20.07	55.05 ± 26.52	37.69 ± 21.90		
	Change between baseline and 12 weeks	-12.20 ± 22.34	-10.69 ± 16.38	-29.32 ± 24.09	0.83	0.06
Pain, mean ± SD, mm	Baseline	27.87 ± 25.83	24.00 ± 28.96	31.84 ± 26.21		
	4 weeks	30.09 ± 25.87	26.32 ± 32.53	30.62 ± 21.25		
	8 weeks	24.94 ± 21.01	23.31 ± 29.98	34.16 ± 25.50		
	12 weeks	26.05 ± 22.35	23.10 ± 29.62	25.78 ± 23.78		
	Change between baseline and 12 weeks	-1.83 ± 23.73	-0.90 ± 26.61	-6.05 ± 29.11	0.92	0.67

SD, standard deviation; VAS, visual analog score.

### 3.3 CTCAE

Peripheral neuropathy severity was assessed using CTCAE grades at baseline and at 4 and 12 weeks. Baseline scores in the placebo, low-dose, and high-dose groups were 1.7 ± 0.6, 1.8 ± 0.6, and 2.0 ± 0.8, respectively. At 12 weeks, the magnitude of change did not differ significantly between the placebo and low-dose groups (−0.1 ± 0.8 *vs*. −0.3 ± 0.6, *p* = 0.32) or between the placebo and high-dose groups (−0.1 ± 0.8 *vs*. −0.4 ± 0.7, *p* = 0.32) (**Supplementary Table S1**).

### 3.4 QoL

**Figure 3:**
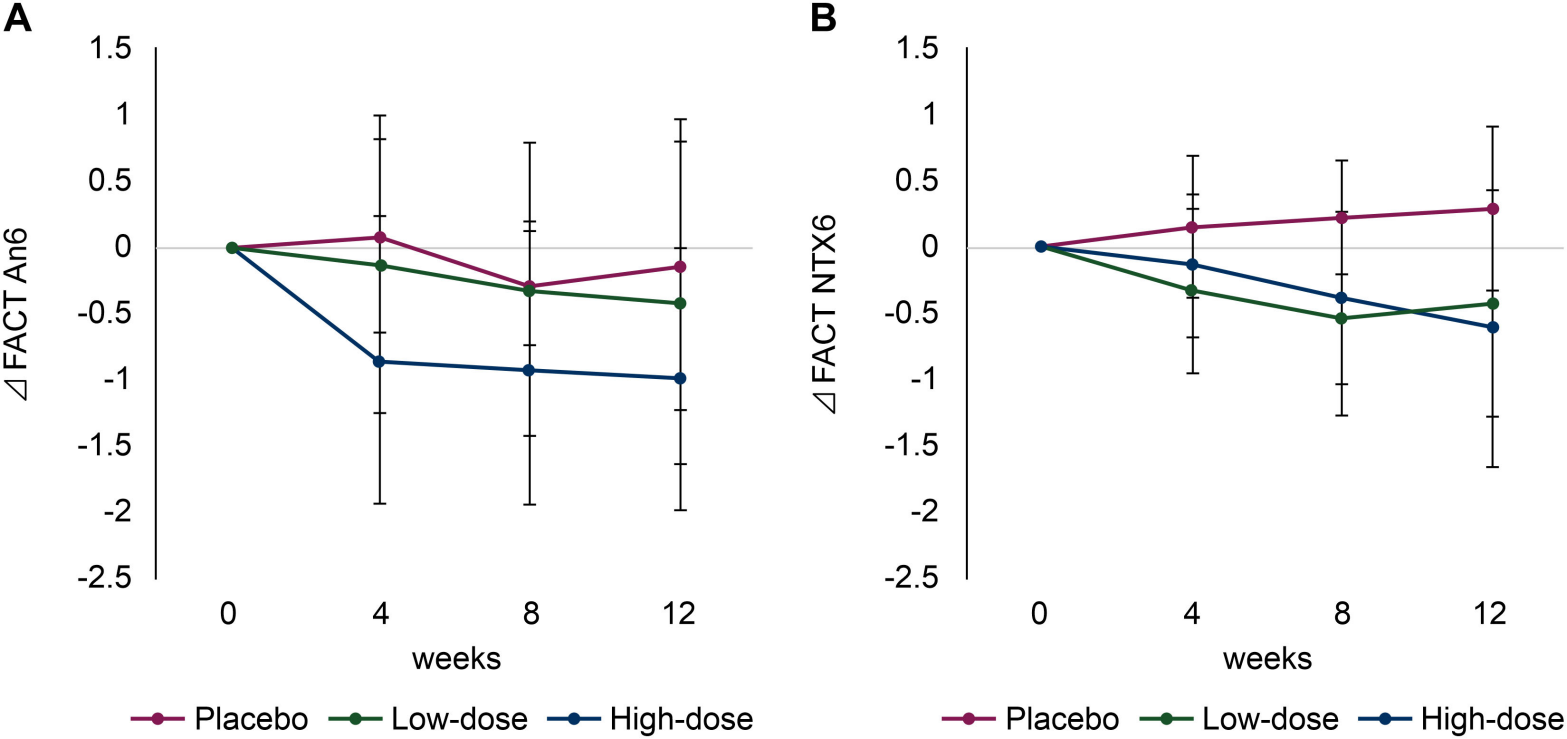
Changes in quality of life (QoL) scores using the Functional Assessment of Cancer Therapy/Gynecologic Oncology Group-Neurotoxicity (FACT/GOG-Ntx). **(A)** Functional Assessment of Cancer Therapy (FACT) An6 (‘difficulty walking’) and **(B)** FACT Ntx6 (‘I have trouble hearing’). Red line: placebo group; green line: low-dose group; blue line: high-dose group.

Among the 40 items of the FACT-GOG/Ntx questionnaire, the An6 (‘difficulty walking’) score in the high-dose group significantly improved compared with that in the placebo group at 12 weeks (−0.14 [95% CI, −1.24 to 0.96] *vs*. −1.00 [95% CI, 0.00 to 2.00]; *p* = 0.02) (**Figure 3A**). Similarly, the Ntx6 (‘I have trouble hearing’) score in the high-dose group was improved compared with that in the placebo group at 12 weeks (−0.29 [95% CI, −0.32 to 0.90] *vs*. −0.62 [95% CI, −1.66 to 0.42]; *p* = 0.01) (**Figure 3B**). A summary of changes in the FACT-GOG/Ntx questionnaire scores is shown in **Supplementary Table S2**.

### 3.5 Adverse events

Serious adverse events directly related to the intervention were not observed. One case of grade 1 anorexia in the low-dose group and one case of grade 2 shiitake dermatitis in the high-dose group were considered causally related but mild; therefore, the patients were allowed to continue treatment. One patient in the placebo group experienced a grade 4 small intestinal obstruction. Adverse events for which a causal relationship with the intervention could not be ruled out included abdominal distention (6.7%), abdominal pain (6.7%), anorexia (6.7%), and skin and subcutaneous tissue disorders (6.7%) in the low-dose group and nausea (6.7%) in the high-dose group (**Supplementary Table S3**).

### 3.6 Adherence to treatment

The median treatment adherence rates were 98.9% (range: 67.0%–100.0%) for the placebo group, 97.7% (range: 75.6%–100.0%) for the low-dose group, and 99.4% (range, 29.1%–100.0%) for the high-dose group.

## 4 Discussion

We explored whether low-dose and high-dose L.E.M. could reduce oxaliplatin-induced numbness experienced by patients undergoing postoperative chemotherapy for CRC. Although no significant difference in the reduction of the VAS numbness scores in the low-dose and placebo groups was observed at 12 weeks—the primary endpoint—a numerical decrease in the VAS numbness score in the high-dose group compared with that in the placebo group was observed. Although no clinically important minimum difference in VAS numbness scores has been established, we observed a change of 17 mm in the high-dose group, which aligned with the findings of a previous systematic review of acute pain that integrated 37 trials and reported a median clinically important minimum difference of 17 mm (8–40 mm) (19). These findings indicate that high-dose L.E.M. extract may have clinical relevance, and that further investigations are warranted.

Oxaliplatin-induced physical damage impairs neuronal function through mechanisms such as DNA damage, voltage-gated ion-channel dysfunction, neuroinflammation, altered transporter function, oxidative stress, mitochondrial dysfunction, and apoptosis (20). Although oxaliplatin is less neurotoxic than cisplatin to dorsal root ganglion neurons because of fewer platinum–DNA adducts, it still induces apoptosis. For oxaliplatin-induced neuropathic pain, the efficacy of duloxetine, which is a serotonin–norepinephrine reuptake inhibitor, has been demonstrated in several randomized trials (18, 21), and a small randomized trial suggested that venlafaxine reduces both acute and chronic neuropathic pain in patients treated with oxaliplatin (22). These agents offer temporary relief from pain associated with CIPN and may enable patients to receive higher cumulative doses of oxaliplatin. However, although options for managing neuropathic pain exist, effective treatments that specifically target numbness in CIPN have not been identified. Moreover, the current agents lack neuroprotective effects and may paradoxically exacerbate OIPN, potentially contributing to motor dysfunction. Consequently, strategies that protect or repair nerve function that can replace analgesic or palliative treatments for OIPN are critically necessary. L.E.M. extract has shown antioxidant and anti-inflammatory properties in preclinical studies, suggesting potential neuroprotective effects (23, 24). Therefore, we focused our investigation on L.E.M. extract as a potential therapeutic agent.

In the high-dose and low-dose groups, adherence to L.E.M. extract treatment was high, and tolerability was acceptable. Serious adverse events causally related to the product were not observed, suggesting minimal safety concerns. Shiitake dermatitis is associated with a toxic or hypersensitivity reaction to lentinan, which is a polysaccharide component of the mushroom cell walls. Lentinan is heat-labile; therefore, inadequately cooked mushrooms often cause such reactions (25). Because L.E.M. extract is prepared via hot water extraction, the incidence of dermatitis may be decreased; however, attention should be focused on skin reactions during ingestion because the exact prevalence of shiitake dermatitis is unknown. In this study, dermatitis was observed in one (6.7%) patient in the low-dose group and one (6.7%) patient in the high-dose group. Participants who were using gabapentin were excluded because shiitake mushroom consumption can increase plasma ergothioneine levels, which stimulates OCTN1-mediated gabapentin secretion, thus potentially reducing blood levels (26). Caution is warranted when using L.E.M. for patients who require prophylactic gabapentin for epilepsy.

These findings indicate that L.E.M. extract may help prevent hearing impairment progression. Common adverse effects of platinum-based drugs include nephrotoxicity and ototoxicity. These drugs interact with DNA, resulting in irreversible changes that prevent tumor cell division. Although oxaliplatin is less ototoxic than cisplatin, sudden hearing loss has occurred during oxaliplatin treatment (27, 28). Both oxaliplatin and cisplatin are toxic to extracochlear hair cells and target thioredoxin reductase in the organ of Corti (29). Oxaliplatin can induce degeneration of hair cells in mouse cochlear tissue fragments through reactive oxygen species accumulation via ferroptosis; however, activation of Nrf2 by resveratrol can reduce cytotoxicity. Future studies should elucidate how L.E.M. extract attenuates these effects.

This study has several limitations. First, the limited follow-up period precluded a thorough evaluation of the long-term effects of L.E.M. extract. Second, the data were obtained exclusively from a Japanese population, which may limit the generalizability of the findings. Therefore, larger studies with longer follow-up periods and more diverse populations are needed to fully elucidate the long-term effects of this agent.

In conclusion, although statistical significance was not observed, high doses of L.E.M. produced a numerically meaningful improvement in numbness scores, suggesting its potential clinical relevance. L.E.M. extract could be a potential neuroprotective agent for managing OIPN and other neurotoxicities. Further studies are necessary to establish its efficacy and explore its use as a neuroprotective agent for patients with cancer and chemotherapy-induced neurotoxicity.

## Data Availability

The raw data supporting the conclusions of this article will be made available by the authors, without undue reservation.

## Glossary

AE: adverse event
CI: confidence interval
CIPN: chemotherapy-induced peripheral neuropathy
CRC: colorectal cancer
CTCAE: Common Terminology Criteria for Adverse Events
FACT/GOG-Ntx: Functional Assessment of Cancer Therapy-Gynecologic Oncology Group-Neurotoxicity
L.E.M.: Lentinula edodes mycelium
OIPN: oxaliplatin-induced peripheral neuropathy
QoL: quality of life
VAS: visual analog scale
